# Combined In Vitro Toxicity and Immunogenicity of Cold Plasma and Pulsed Electric Fields

**DOI:** 10.3390/biomedicines10123084

**Published:** 2022-11-30

**Authors:** Christina M. Wolff, Juergen F. Kolb, Sander Bekeschus

**Affiliations:** 1 ZIK Plasmatis—Leibniz Institute for Plasma Science and Technology (INP), Felix-Hausdorff-Str. 2, 17489 Greifswald, Germany; 2 Institute of Physics, University of Rostock, Albert-Einstein-Str. 23-24, 18059 Rostock, Germany

**Keywords:** apoptosis, kINPen, leukemia, plasma medicine, reactive oxygen species, ROS

## Abstract

In modern oncology, therapies are based on combining monotherapies to overcome treatment resistance and increase therapy precision. The application of microsecond-pulsed electric fields (PEF) is approved to enhance local chemotherapeutic drug uptake within combination electrochemotherapy regimens. Reactive oxygen species (ROS) have been implicated in anticancer effects, and cold physical plasma produces vast amounts of ROS, which have recently been shown to benefit head and neck cancer patients. PEF and cold plasma technology have been linked to immunogenic cell death (ICD) induction, a regulated cell death accompanied by sterile inflammation that promotes antitumor immunity. To this end, we investigated the combined effect of both treatments regarding their intracellular ROS accumulation, toxicity, ICD-related marker expression, and optimal exposure sequence in a leukemia model cell line. The combination treatment substantially increased ROS and intracellular glutathione levels, leading to additive cytotoxic effects accompanied by a significantly increased expression of ICD markers, such as the eat-me signal calreticulin (CRT). Preconditioned treatment with cold plasma followed by PEF exposure was the most potent treatment sequence. The results indicate additive effects of cold plasma and PEF, motivating further studies in skin and breast tumor models for the future improvement of ECT in such patients.

## 1. Introduction

Cold physical plasmas are partially ionized gases that have ion temperatures close to the body temperature when applied in plasma medicine [1]. Plasma generates UV (ultraviolet) radiation, visible light, electromagnetic fields, thermal radiation, electrons and ions, and reactive oxygen and nitrogen species (ROS/RNS) [2]. Due to these multi-component effectors, studying plasma processes for (bio)technology applications has been a highly active field of research [3]. For instance, plasma treatment of enzymes deposited on glass enhanced enzyme activity by a factor of seven [4]. Other studies have shown plasma to play a critical role in biosensor optimization in environmental samples [5] and in improving pharmaceutical processes [6]. For treating eukaryotic cells in vitro, ROS/RNS were identified as the main mediators of plasma effects [7,8]. In clinical applications, plasma is used to treat chronic ulcers [9,10,11] by decreasing microbial burden in wounds [12,13,14], altering inflammation [15], and increasing oxygenation [16,17,18]. Many studies focus on its highly toxic effect on cancer cells. Plasma treatment responses found in cancer cell lines are apoptosis, growth inhibition, cell cycle arrest, and cytoskeletal and mitochondrial damage [19]. Anticancer action of plasma has been shown across many tumor cell lines of various tumor entities [20,21]. Plasma was shown to be active against tumor growth, such as melanoma, in mice in vivo [22]. Moreover, plasma was shown to induce immunogenic cell death (ICD) [23] and, hence, has great potential to support antitumor immunity, especially within individualized combination treatment regimens. The importance of other cell types, such as immune and stromal cells, is becoming increasingly recognized in oncology and plasma oncology research [23,24].

Pulsed electric fields (PEFs) are frequently used for cancer treatment within electrochemotherapy [25]. In this application, pulse durations in the range of microseconds are employed, which predominantly affect cellular plasma membranes, causing permeabilization. In combination with chemotherapeutics, bleomycin and cisplatin are commonly utilized. The permeabilized cell membranes allow efficient uptake of cytotoxic drugs. Nevertheless, PEFs in the microsecond range can also generate toxic cell effects by either irreversible electroporation or intracellular cell death signaling, which are processes shown to include ROS formation [26].

To this end, we combined both technologies in vitro using leukemia cells as a model system to investigate their potential to provide additive effects concerning intracellular ROS accumulation, cytotoxicity, and increased surface markers associated with ICD, identifying overall promising results of this combination treatment.

## 2. Materials and Methods

### 2.1. Cell Culture

The human lymphoma cell line Jurkat (ACC282) and the human lymphoblast cell line TK6 (ATCC CRL-8015) were used. The cells were cultured in Dulbecco’s Modified Eagle’s Medium (DMEM; PAN-Biotech, Aidenbach, Germany) or Roswell Park Memorial Institute (RPMI) 1640 medium (PAN-Biotech, Aidenbach, Germany), each containing 10% fetal bovine serum (Sigma-Aldrich-Chemie, Taufkirchen, Germany), 1% glutamine (PAN-Biotech), and 1% penicillin/streptomycin (PAN-Biotech, Aidenbach, Germany) at 37 °C and 5% CO_2_. Before the treatment, the cells were seeded at 5 × 10^4^ in 100 µL DMEM into 96-well round bottom plates (Thermo Fisher Scientific, Dreieich, Germany). For a different set of experiments, 2.5 × 10^5^ cells in 500 µL of RPMI medium were seeded into flat-bottom 24-well plates (Eppendorf, Hamburg, Germany).

### 2.2. Application of Pulsed Electric Fields and Cold Physical Plasma

The atmospheric pressure plasma jet kINPen (neoplas MED, Greifswald, Germany) was utilized as a plasma source. The plasma jet was operated at two standard liters per minute using argon gas and at a deposited power of 1 W and a voltage of 2–6 kV_pp_. The kINPen is the most completely characterized cold plasma source worldwide, based on the literature available on its physical characterization and biomedical and clinical effects [27,28]. The plasma exposure of the samples was done only once (single exposure). Plasma treatment times were pre-optimized depending on the cell type and assay used, and were found to be optimal (i.e., a modest but visible effect) for Jurkat cells at 40 s and TK.6 cells at 7 s in the 96-well treatment regimen. For the application of pulsed electric fields (PEFs), a commercially available electro square porator (ECM 830; BTX Havard Apparatus, Holliston, MA, USA) was utilized. PEF pulse duration was 100 µs or 10 µs, and electric field strengths were between 0.7 kV/cm and 1.4 k/cm, depending on the experiment. PEF was applied as eight consecutive pulses at 1 Hz. For the PEF treatment, the cells were transferred into 2 mm electroporation cuvettes (Biozym Scientific, Oldendorf, Germany). To compare the PEF single application to the combination treatment of plasma first and PEF second in the 96-well setup, the cells were seeded into the plate first and then pooled into the electroporation cuvettes.

### 2.3. Cell Viability

For the discrimination between viable and dead cells, 4’6-diamidino-2-phenylindole (DAPI, 1 µM; Sigma-Aldrich, St. Louis, MO, USA) and CellEvent Caspase-3/7 Green Detection Reagent (1 µM; Thermo Fisher Scientific, Waltham, NJ, USA) were utilized. After 15 min of incubation at 37 °C, the percentage of each cell population staining negative or single- or double-positive was determined using flow cytometry (CytoFLEX S; Beckman-Coulter, Krefeld, Germany) and Kaluza 2.1.3 analysis software (Beckman-Coulter, Krefeld, Germany).

### 2.4. Intracellular Reactive Oxygen Species Analysis

To assess the intracellular oxidation, the cells were stained with DAPI and BODIPY 581/591 C11 (1 µM; Thermo Fisher Scientific, Waltham, NJ, USA) for lipid peroxidation, MitoSox Red (1 µM; Thermo Fisher Scientific, Waltham, NJ, USA) for mitochondrial superoxide, and CM-H_2_DCF-DA (1 µM; Thermo Fisher Scientific, Waltham, NJ, USA) for cytosolic reactive oxygen species (ROS). The staining was performed for 15 min at 37 °C in phosphate–buffer saline (PBS; PAN-Biotech, Aidenbach, Germany). Afterward, the cells were washed, resuspended in fully supplemented DMEM medium, and treated as described above. Immediately after the treatment, the samples were measured using flow cytometry.

### 2.5. Glutathione Content and Mitochondrial Membrane Potential

For the measurement of mitochondrial membrane potential (ΔΨ_m_), MitoTracker Red FM Dye (1 µM; Thermo Fisher Scientific, Waltham, NJ, USA) was employed. The cells were stained with DAPI and the MitoTracker in PBS for 15 min. To determine the glutathione content, the cells were stained with propidium iodide (PI; 5 µg/mL; Santa Cruz Biotechnology, Heidelberg, Germany) and ThiolTracker Violet (10 µM; Thermo Fisher Scientific, Waltham, NJ, USA) in PBS containing calcium and magnesium. The staining was executed for 30 min at 37 °C. After the staining, the cells were washed with RPMI, and the fluorescence was measured using flow cytometry at 4 h and 24 h after the treatment.

### 2.6. Antibody Staining of Surface Markers

The following antibodies were utilized to analyze cell surface markers: CD47 conjugated with brilliant violet 605 (BV605; BioLegend, Amsterdam, The Netherlands), CD274 conjugated with BV650 (BioLegend, Amsterdam, The Netherlands), heat shock protein 90 (HSP90) conjugated with phycoerythrin (PE; Enzo Life Science, Lörrach, Germany), and calreticulin (CRT) conjugated with Alexa Fluor 647 (Bio-Techne, Wiesbaden, Germany). Twenty-four hours after the treatment, the cells were first washed with autoMACS Running Buffer (Miltenyi Biotec, Bergisch Gladbach, Germany) and subsequently stained with the antibodies mentioned above and DAPI (1 µM) in Running Buffer (100 µL) for 30 min at room temperature. After two washing cycles, the samples were measured using flow cytometry.

### 2.7. Analysis of Plasma-Treated Liquid

ROS and RNS were profiled in plasma-treated liquids (100 µL of PBS in a 96-well round bottom plate) by utilizing Amplex Ultra Red (Thermo Fisher Scientific, Waltham, NJ, USA) and the Griess assay (Thermo Fisher Scientific, Waltham, NJ, USA), as described previously [29]. The temperature of plasma-treated liquid was analyzed using an infrared thermometer to monitor the liquid temperature development.

### 2.8. Statistical Analysis

For each assay, three independent experiments, each with at least two technical replicates, were performed and included in the data analysis. Data were analyzed and graphed using prism 9.4.1 (GraphPad Software, San Diego, CA, USA). One-way ANOVA with Turkey’s multiple comparison test was executed to determine the degree of statistical significance between the different groups. The level of significance is indicated as follows: *p* < 0.05 (*), *p* < 0.01 (**), and *p* < 0.001 (***).

## 3. Results

**Figure 1:**
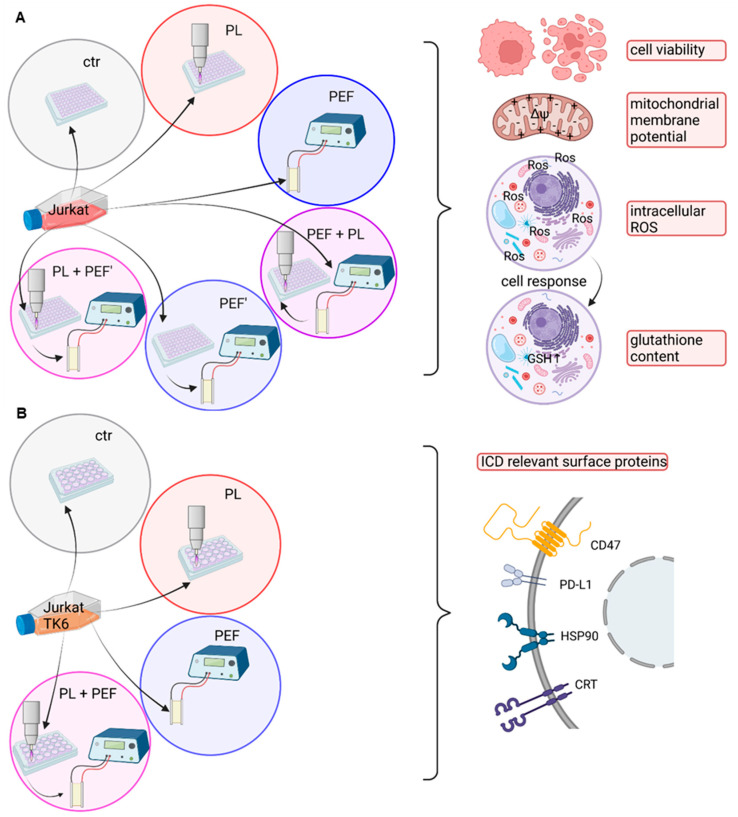
Experimental plasma and PEF combination set up in 96- or 24-well plate. Jurkat cells were utilized to determine the cell viability, mitochondrial membrane potential, intracellular ROS, and glutathione content. The experiments were performed in DMEM and 96-well plates. The sequence order of plasma first and PEF second and vice versa were investigated (**A**). For the ICD analysis, Jurkat and TK6 cells were seeded in a 24-well plate in RPMI. The ICD-relevant surface proteins, CD47, PD-L1, HSP90, and CRT, were measured via flow cytometry. In the combination, the treatment order of plasma first and PEF second was examined (**B**). ctr: control; PL: plasma; PEF: pulsed electric fields; PEF’: cells were seeded into 96 well plates before being transferred into cuvettes; ΔΨ: mitochondrial membrane potential; ROS: reactive oxygen species; GSH: glutathione; CD47: cluster of differentiation 47; PD-L1: programmed cell death ligand 1; HSP90: heat shock protein 90; CRT: calreticulin; ICD: immunogenic cell death.

In the following study, the plasma treatments were performed in either a 96-well plate and DMEM for the redox analysis (Figure 1A) or a 24-well plate and RPMI for the ICD investigation (Figure 1B). The cells were transferred into a cuvette for the pulsed electric field (PEF) treatments. In the case of the 96-well plate, four wells were pooled into one cuvette, leading to a significant cell loss. This caused differences in the toxicity of the PEF treatment. To compare the single treatment with the combination treatment, the PEF-treated cells were seeded into a 96-well plate before being transferred to the cuvette when plasma was applied first. Due to the experimental setup, the time between the plasma and the PEF treatment was 15 min when the cells were seeded in 96-well plates. In the ICD experiments, PEF was applied immediately after the plasma regimen.

### 3.1. Dependent on the Treatment Regimen, the Combination of Plasma and PEF Amplifies Cell Toxicity

**Figure 2:**
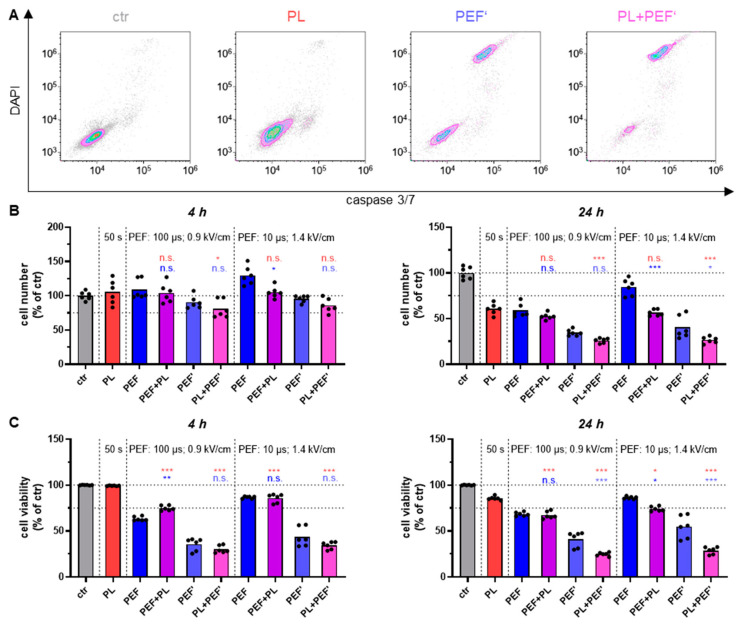
Combination effect of different plasma and PEF treatments on Jurkat cells in DMEM regarding cell viability and number. The cell viability was measured by DAPI and CellEvent Caspase-3/7 Green Detection Reagent using flow cytometry after 4 h and 24 h (**A**,**C**). Concurrently, the cell number was determined (**B**). Data are shown as one representative (**A**) or mean from three independent experiments with several replicates each (**B**,**C**). One-way ANOVA with Turkey’s multiple comparison test was performed (*: *p* < 0.05, ** *p* < 0.01 and ***: *p* < 0.001). ctr: control; PL: plasma; PEF: pulsed electric field; PEF’: cells were seeded into 96-well plates before being transferred into cuvettes; n.s.: not significant.

Cell viability and cell number were measured via flow cytometry after 4 h and 24 h to determine the cell toxicity. To assess overall cell viability, the cells were stained for apoptosis (active caspases 3 and 7) and terminal cell death (DAPI). Accordingly, the caspase 3/7 positive cells are the early apoptotic population, the DAPI positive cells are the necrotic population, the double-positive cells are the late apoptotic population, and the double-negative cells are the viable cells (Figure 2A). The cell count after 4 h revealed barely any effects, whereas the cell viability of PEF and combined treated cells were already significantly reduced (Figure 2B,C). Nevertheless, a combination effect was not observed because there was no significant reduction in the combination-treated cells compared to the PEF-treated ones. After 24 h, a significant decrease in the cell number and viability was shown for almost all application regimens (Figure 2B,C). The combination was most efficient when plasma was applied before PEF. Regarding cell viability, plasma and PEF increased their cell toxic effects when plasma was applied first, independent of the PEF settings. In relation to cell numbers, only the application order of plasma first and PEF (10 µs and 1.4 kV/cm) second significantly augmented the cytotoxicity.

### 3.2. Mitochondrial Membrane Potential Correlates with Cell Toxicity

**Figure 3:**
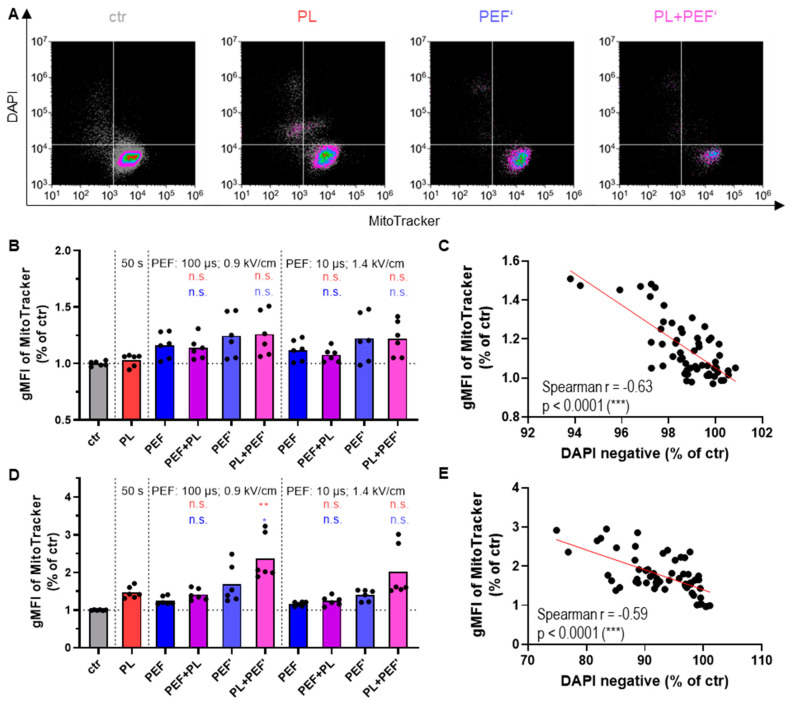
Mitochondrial membrane potential of Jurkat cells in DMEM after plasma and PEF treatment. MitoTracker Red FM was used to analyze all cells’ mitochondrial membrane potential plotted against the DAPI signal after 24 h (**A**). The mitochondrial membrane potential of the viable cells is displayed after 4 h (**B**) and 24 h (**D**). Data are shown as one representative (**A**) or mean from three independent experiments with several replicates each (**B**,**D**). The analysis for mitochondrial membrane potential in relation to the DAPI negative cells was done using nonparametric, two-tailed Spearman correlation with 95% confidence intervals for the 4 h (**C**) and 24 h (**E**) measurements. The fluorescence intensity of the dyes was evaluated via flow cytometry. One-way ANOVA with Turkey’s multiple comparison test was performed (*: *p* < 0.05, **: *p* < 0.01). ctr: control; PL: plasma; PEF: pulsed electric field; PEF’: cells were seeded into 96-well plates before being transferred into cuvettes; n.s.: not significant; gMFI: fluorescence intensity of the geometric mean.

The mitochondrial membrane potential (ΔΨ_m_) generated by proton pumps is essential to maintain oxidative phosphorylation and plays a key role in mitochondria homeostasis [30]. The critical step of apoptosis is irreversible and widespread mitochondrial outer membrane permeabilization, leading to the release of mitochondrial intermembrane space proteins, such as cytochrome c, into the cytosol [31]. This leads ultimately to a decrease of ΔΨ_m_ [32]. Therefore, the ΔΨ_m_ was determined after 4 h and 24 h, at the same time as the cell viability (Figure 3A). MitoTracker Red FM dye accumulates into the mitochondrial membrane, dependent on the ΔΨ_m_. Its fluorescence intensity in viable cells was augmented in most treatment groups after 4 h (Figure 3B) and all groups at 24 h (Figure 3D). As expected, when the MitoTracker signal was plotted against the DAPI signal, it revealed that the DAPI-positive (terminally dead) cells displayed a lower MitoTracker fluorescence intensity (Figure 3A). Nevertheless, the MitoTracker signal negatively correlated with cell viability (Figure 3C,E). This finding might be because mitochondria undergo an initial priming phase associated with hyperpolarization during apoptosis. This leads to an effector phase, during which mitochondria swell and release cytochrome c [33]. Hence, the cells displaying an increased MitoTracker signal are probably dying cells in an early apoptotic phase.

### 3.3. PEF Induces Intracellular ROS Increase

**Figure 4:**
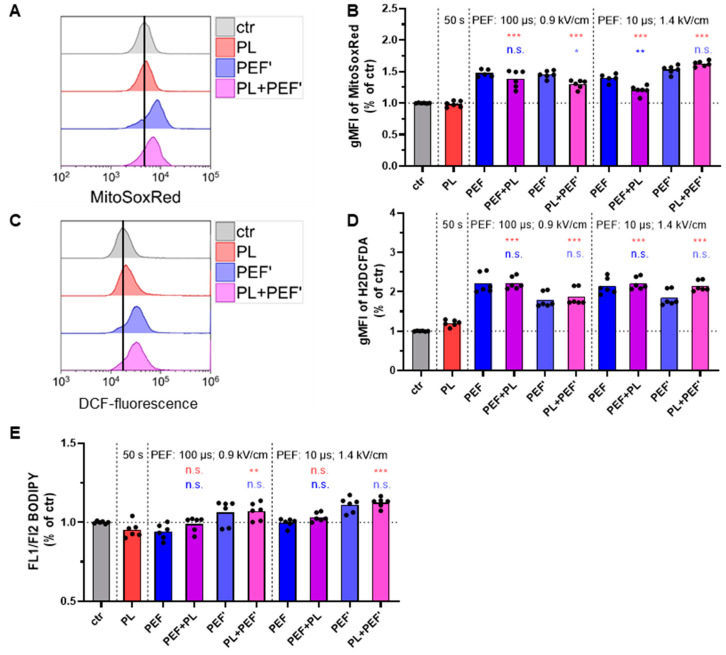
Intracellular ROS in Jurkat cells directly after applying plasma and PEF. The ROS level was analyzed via MitoSOX Red (**A**,**B**), indicating mitochondrial superoxide CM-H2DCFDA (**C**,**D**), indicating cytosolic ROS, and BODIPY 581/591 C11, indicating lipid peroxidation (**E**). Data are shown as one representative (**A**,**C**) or mean from three independent experiments with several replicates each (**B**,**C**,**E**). One-way ANOVA with Turkey’s multiple comparison test was performed (*: *p* < 0.05, **: *p* < 0.01, and ***: *p* < 0.001). ctr: control; PL: plasma; PEF: pulsed electric field; PEF’: cells were seeded into 96-well plates before being transferred into cuvettes; n.s.: not significant; gMFI: fluorescence intensity of the geometric mean; FL: fluorescence channel.

**Figure A1:**
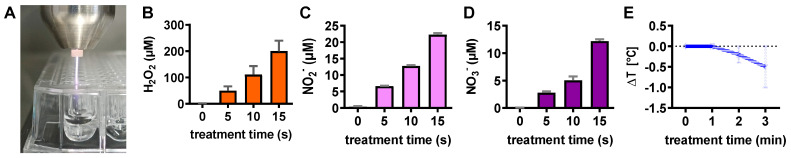
Plasma-liquid analysis. (**A**) treatment setup using the atmospheric plasma argon plasma jet kINPen exposing PBS; (**B**–**D**) quantification of H_2_O_2_ (**B**), nitrite (**C**), and nitrate (**D**) in plasma-treated PBS; (**E**) temperature changes (Δ) of plasma-treated liquid normalized to untreated liquid.

To further analyze the effect on mitochondria directly after plasma and PEF treatment, MitoSOX Red was used to measure the superoxide anion (Figure 4A,B) generated as a byproduct of oxidative phosphorylation and the predominant ROS in mitochondria [34]. The superoxide indicator is active in live cells, cell membrane permeable, and selectively targets the mitochondria. Once oxidized by superoxide, it becomes highly fluorescent upon binding to nucleic acids [35]. Furthermore, the fluorescence intensity of DCF was measured to determine the cytosolic ROS (Figure 4C,D). The ROS indicator passively diffuses into cells, where intracellular esterases cleave its acetate group, enabling its chloromethyl group to react with intracellular glutathione and other thiols [36]. Subsequent oxidation yields a fluorescent adduct that can be quantified via flow cytometry. The first barrier of the cell that can be affected by plasma-generated ROS is the cell membrane consisting of a lipid bilayer. Hence, lipid peroxidation was determined by BODIPY 581/591 C11 staining (Figure 4E), which is lipophilic and therefore incorporated into the cell membrane. Upon oxidation, the fluorescence emission peak shifts from ~590 nm to ~510 nm [37]. There was little increase in lipid peroxidation compared to the control (Figure 4E). By contrast, cytosolic ROS were significantly increased for the PEF and combined treated cells. Nevertheless, there was no augmentation when plasma and PEF were applied, compared to the single PEF-treated cells (Figure 4C,D). Likewise, the mitochondrial superoxide level was highly enhanced in all PEF-treated cells within single and combined regimens. A further increase in the combination treatment was only found when plasma was applied first (Figure 4A,B). The results indicate that PEF induces a much higher oxidative stress directly after the treatment than plasma did in our setup. The occurrence of oxidative stress is due to supraphysiological levels of reactive species. To confirm their trajectories from the plasma jet to the liquid in which the cells resided, reactive species were analyzed in plasma-treated PBS (Figure A1A). Hydrogen peroxide (Figure A1B), nitrite (Figure A1C), and nitrate (Figure A1D) were found, and their concentrations increased in a plasma treatment time-dependent fashion. By contrast, temperature effects were not present, as plasma jet treatment of liquids did not increase the temperature (Figure A1E).

### 3.4. The Glutathione Increases to Counterbalance Oxidative Stress

**Figure 5:**
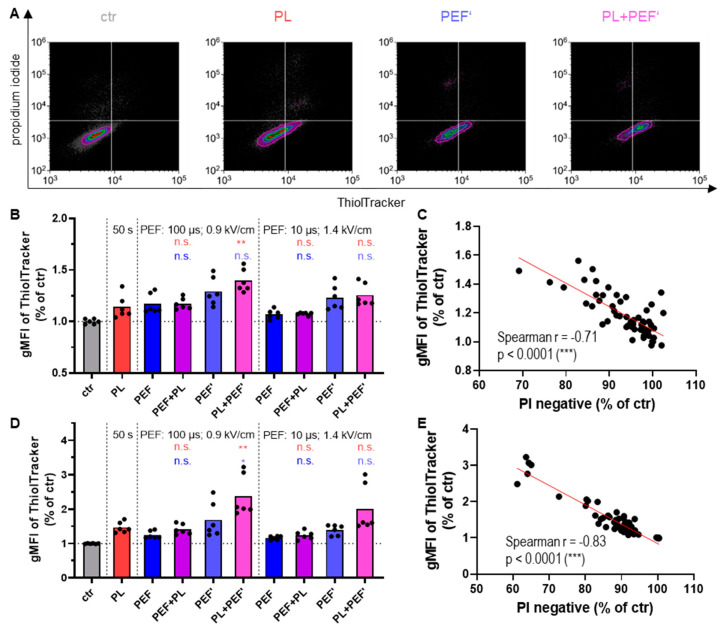
Thiol content of Jurkat cells in DMEM after plasma and PEF treatment. The thiol content of the viable cells was determined by ThiolTracker Violet after 4 h (**B**) and 24 h (**A**,**D**). Concurrently, the cell viability was measured via PI. Data are shown as one representative (**A**) or mean from three independent experiments with several replicates each (**B**,**D**). The analysis for total thiol content in relation to the PI negative cells was performed using nonparametric, two-tailed Spearman correlation with 95% confidence intervals after 4 h (**C**) and 24 h (**E**). The fluorescence intensity of the dyes was evaluated via flow cytometry. One-way ANOVA with Turkey’s multiple comparison test was performed (*: *p* < 0.05 and **: *p* < 0.01). ctr: control; PL: plasma; PEF: pulsed electric field; PEF’: cells were seeded into 96-well plates before being transferred into cuvettes; ns: not significant; gMFI: fluorescence intensity of the geometric mean.

Glutathione is a tripeptide and the major antioxidant inside the cell [38]. The glutathione content (Figure 5A) of the viable cells was determined after 4 h (Figure 5B) and 24 h (Figure 5D) using the fluorescent dye ThiolTracker Violet, which reacts actively with reduced thiols in intact cells. Therefore, it can be used to estimate the cellular level of reduced glutathione [39]. The glutathione content increased strongly in the combined treatment when plasma was applied before PEF (Figure 5B,D), corresponding to the most strongly decreased cell viability (Figure 2B,C). Accordingly, there was a strong negative correlation between the glutathione content and the PI-negative cells at 4 h (Figure 5C) and 24 h (Figure 5E). This suggests that the cells attempt to counterbalance the toxic oxidative stress (Figure 4) by increasing thiol group-containing antioxidants.

### 3.5. Principal Component Analysis of Cell Toxicity, Intracellular ROS, Glutathione Content, and Mitochondrial Membrane Potential of the Different Treatment Groups

**Figure 6:**
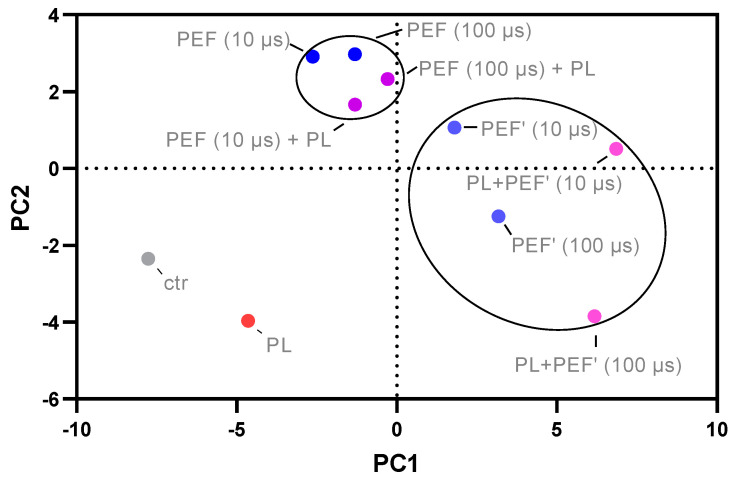
Principal component (PC) analysis of cell viability and number, intracellular ROS, thiol content, and mitochondrial membrane potential. The treatment order of plasma first and PEF second varied the most from the single applications.

Principal component analysis (PCA) was performed to detect which treatment groups vary or match the most (Figure 6). Interestingly, the combination treatment of PEF first and plasma second was very similar to the PEF single applications, regardless of the pulse length. Conversely, if plasma was applied before PEF, the treatment groups differentiated from the only PEF treatment regarding PC1 (pulse length: 10 µs) or PC2 (pulse length: 100 µs). The single plasma application varied significantly from all combined treatments. It was concluded that the combination order of plasma after PEF should be evaluated further. Hence, it was selected for the following analysis regarding the ICD markers.

### 3.6. Immunological Cell Death Markers in Jurkat and TK6 Cells after Plasma and PEF Treatment

**Figure 7:**
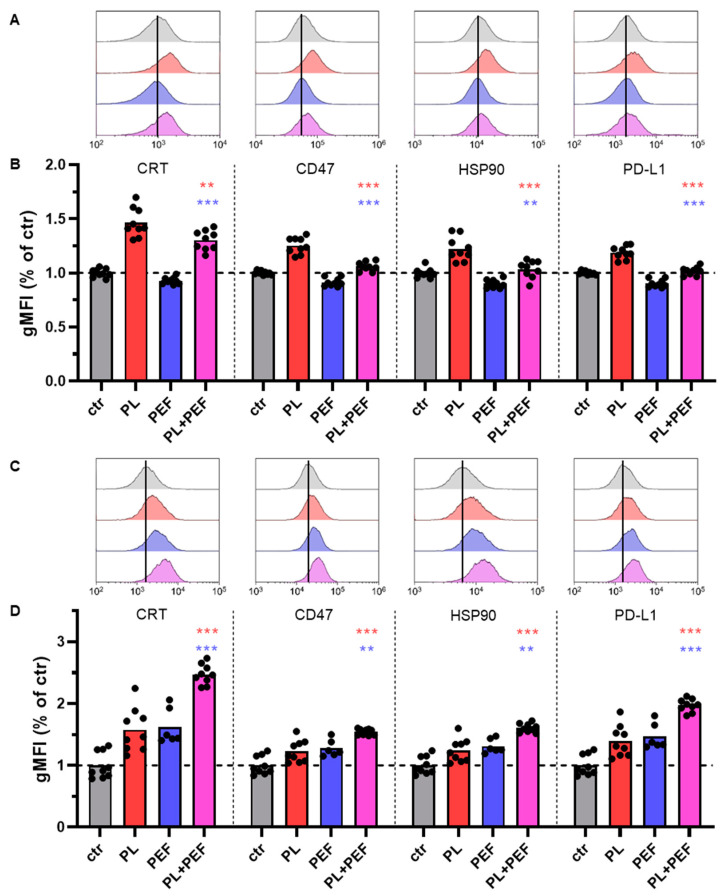
Effects on ICD markers of Jurkat and TK6 cells in RPMI after plasma and PEF treatment. The effect on the cell surface markers CRT, CD47, HSP90, and PD-L1, indicating ICD, was analyzed 24 h after treating Jurkat (**A**,**B**) and TK6 (**C**,**D**) cells. Data are shown as one representative (**A**,**C**) or mean from three independent experiments with several replicates each (**B**,**D**). One-way ANOVA with Turkey’s multiple comparison test was performed (**: *p* < 0.01 and ***: *p* < 0.001). ctr: control; PL: plasma; PEF: pulsed electric field; ns: not significant; CD47: cluster of differentiation 47; PD-L1: programmed cell death ligand 1; HSP90: heat shock protein 90; CRT: calreticulin.

The experimental setup was slightly changed to investigate immunological cell death (ICD) after the combination treatment of plasma and PEF. A 24-well plate was utilized to resolve the issue of cell loss when transferring the cells from the plate to the cuvette. Furthermore, RPMI medium was used, so the plasma treatment was more effective. Instead of two different pulse lengths, only the pulse length of 100 µs was applied, which is also the pulse length used in electrochemotherapy [40]. The investigations were performed in two cell lines, the human T-lymphoma Jurkat cell line and the human lymphoblastoid TK6 cell line. The surface expression was determined for the following proteins: the damage-associated molecular patterns (DAMPs) calreticulin (CRT) and heat shock protein (HSP) 90 as well as the immune checkpoint inhibitor, programmed cell death ligand 1 (PD-L1), and the *don’t eat me* signal to macrophages cluster of differentiation 47 (CD47) (Figure 7). All four surface markers were significantly increased after plasma treatment for the Jurkat cells. By contrast, the expression was significantly reduced after PEF application compared to the control (Figure 7A,B). Interestingly, cell viability was much less affected in the PEF treatment group than in the plasma treatment group. For the combined treated cells, the expression of PD-L1, CD47, and HSP90 was quite similar to the control. Although CRT was significantly induced compared to the control, it decreased compared to the plasma group. Conversely, the expression of ICD markers was considerably augmented for all three treatment applications in TK6 cells (Figure 7C,D). After the combined application, cell viability was significantly lower and marker expression significantly higher than in the single treatments.

## 4. Discussion

The idea of combining plasma and PEFs was originally based on the following hypotheses: (1) plasma-treated cells are easier to electroporate due to their oxidation status, (2) electroporated membranes allow the easier entrance of plasma-mediated ROS, and (3) the intracellular cell death signaling cascades induced by plasma and PEFs augment each other. A previous study using a similar setup has already suggested that an enhancement of irreversible electroporation is not the main reason for an increased cell death effect after the combined treatment [41]. In our experiment, we did not observe a significant increase in lipid peroxidation after plasma treatment. Hence, the first hypothesis seems to be improbable. Regarding the second hypothesis, the augmentation of glutathione 24 h after treatment suggested a cell stress response against intracellular ROS. Nevertheless, ROS in the combination approaches was barely increased, compared to the single PEF applications, directly after the treatment. Therefore, the cell stress response against an elevated ROS level might be induced by ROS-based cell signaling cascades [42]. The negative correlation between glutathione level and cell viability supports this further. Thus, our third hypothesis about the intracellular cell death signaling cascades seems most likely.

Previous studies combining PEF plasma-treated PBS or medium showed the potential for additive cytotoxicity. However, evidence suggests the treatment order to be of relevance. For instance, adding plasma-treated medium first, followed by microsecond PEF, was more cytotoxic than vice versa [43]. For plasma-treated PBS, the combined application completely abolished the tumor spheroid growth, supposedly by enabling the plasma-generated ROS to reach the spheroid core [44]. It was also found that plasma-treated PBS reduces the PEF amplitude required for cell membrane electropermeabilization [45]. Using direct plasma treatment, one study found increased inactivation of pancreatic cancer cells and an augmented efficiency of gene electrotransfer by combining PEF in the nanosecond range and nanosecond pulsed plasma jets [46]. Hence, the experimental approach can be modulated so that the electroporation or ROS entrance into the cell is facilitated by plasma pretreatment. Moreover, a previous study reported that both treatment orders are effective if applied directly after each other [41]. Nevertheless, in our setup, the plasma pretreatment was much more effective, whereas the PEF pretreatment gave similar results as the PEF single treatment (Figure 6).

Several cell death mechanisms have been described for plasma-treated cancer cells, including apoptosis, autophagy, pyroptosis, and ferroptosis [47]. Plasma-induced apoptosis can be executed by different signaling pathways, including intrinsic or mitochondrial cell death signaling and extrinsic apoptotic pathway signaling through cell death receptors [48,49]. Autophagy is the process of eliminating dysfunctional or damaged cell organelles. Plasma-mediated ROS can increase autophagosome formation, inducing autophagic cell death [50]. Pyroptosis has some characteristics of apoptosis as well as necrosis [51]. Interestingly, it is an inflammatory form of cell death, and it has been demonstrated that plasma treatment induces ICD by generating ROS [52]. Likewise, ferroptosis can provoke an immune response. One of its hallmarks is excessive membrane lipid peroxidation [53,54]. Similar to plasma, PEF has also been shown to promote several cell death signaling pathways, depending on the experimental setup, including apoptosis, necrosis, necroptosis, and pyroptosis [55]. In this context, PEF has been described as acting on mitochondria directly and indirectly by changing membrane potentials and—through intracellular cytochrome C release—inhibiting ATP production, respectively [56]. Necrosis and necroptosis can trigger immunity [57,58]. Because ICD after cancer treatments is crucial for eradicating tumors on a long-term scale to prevent disease recurrence, we investigated the effect of single and combination treatment on several ICD markers. CRT and HSP90 are well-known DAMPs; hence, their upregulation indicates ICD [59]. Inside the endoplasmic reticulum (ER), CRT performs two major functions, chaperoning and regulation of calcium homeostasis [60]. On the cell surface, it facilitates the phagocytic uptake of apoptotic and cancer cells [61]. Conversely, CD47 is a cell surface marker, well recognized for its anti-phagocytic function [62]. Moreover, the glycoprotein is associated with chemoresistance and cell metastasis [63]. Although the *eat-me* signal CRT was decreased after the combination treatment compared to the plasma group in Jurkat cells, the anti-phagocytic marker was also reduced. Likewise, the PD-L1 level, which has an immune-suppressive effect [64], declined in the combined group. Hence, we found some indications for ICD after the combination of plasma and PEF in Jurkat cells. In contrast to the Jurkat cells, the TK6 cell lines displayed increased surface proteins after both single and combination treatments. The marker expression in the combination group was significantly higher than in the plasma and PEF groups. While the level of CD47 and PD-L1 doubled in the combination treatment, the level of HSP90 increased in parallel. HSP90 exposure attracts the attention of the innate immune system when translocated to the membrane [59]. The expression of the phagocytic signal CRT was augmented even more, indicating that the immunogenic response was at least similar, if not better, than in the single treatment groups.

## 5. Conclusions

Our hypothesis that either plasma-mediated ROS facilitate the electroporation of the oxidized cell membrane or that the electroporated membrane enhances the cellular uptake of the ROS could not be confirmed. Nevertheless, the cytotoxicity in the plasma-pretreated combination group was increased, suggesting the enhancement of intracellular signaling cascades. At the same time, the ICD induced by the single treatment does not seem to be reduced, and possibly even slightly increased, in the combination approach. In vivo experiments could be performed to confirm our conclusion.

## Data Availability

The data of this manuscript can be retrieved from the corresponding author upon reasonable request.
